# Protons in small spaces: Discrete simulations of vesicle acidification

**DOI:** 10.1371/journal.pcbi.1007539

**Published:** 2019-12-23

**Authors:** Apeksha Singh, Frank V. Marcoline, Salome Veshaguri, Aimee W. Kao, Marcel Bruchez, Joseph A. Mindell, Dimitrios Stamou, Michael Grabe

**Affiliations:** 1 College of Letters and Science, University of California Berkeley, Berkeley, California, United States of America; 2 Cardiovascular Research Institute, Department of Pharmaceutical Chemistry, University of California San Francisco, San Francisco, California, United States of America; 3 Bionanotecnology and Nanomedicine Laboratory, University of Copenhagen, Copenhagen, Denmark; 4 Department of Chemistry, University of Copenhagen, Copenhagen, Denmark; 5 Nano-Science Center, University of Copenhagen, Copenhagen, Denmark; 6 Lundbeck Foundation Center Biomembranes in Nanomedicine, University of Copenhagen, Copenhagen, Denmark; 7 Memory and Aging Center, Department of Neurology, University of California San Francisco, San Francisco, California, United States of America; 8 Department of Chemistry, Carnegie Mellon University, Pittsburgh, Pennsylvania, United States of America; 9 Department of Biological Sciences, Carnegie Mellon University, Pittsburgh, Pennsylvania, United States of America; 10 Molecular Biosensor and Imaging Center, Carnegie Mellon University, Pittsburgh, Pennsylvania, United States of America; 11 Membrane Transport Biophysics Unit, National Institute of Neurological Disorders and Stroke, National Institutes of Health, Bethesda, Maryland, United States of America; University of Chicago, UNITED STATES

## Abstract

The lumenal *p*H of an organelle is one of its defining characteristics and central to its biological function. Experiments have elucidated many of the key *p*H regulatory elements and how they vary from compartment-to-compartment, and continuum mathematical models have played an important role in understanding how these elements (proton pumps, counter-ion fluxes, membrane potential, buffering capacity, etc.) work together to achieve specific *p*H setpoints. While continuum models have proven successful in describing ion regulation at the cellular length scale, it is unknown if they are valid at the subcellular level where volumes are small, ion numbers may fluctuate wildly, and biochemical heterogeneity is large. Here, we create a discrete, stochastic (DS) model of vesicular acidification to answer this question. We used this simplified model to analyze *p*H measurements of isolated vesicles containing single proton pumps and compared these results to solutions from a continuum, ordinary differential equations (ODE)-based model. Both models predict similar parameter estimates for the mean proton pumping rate, membrane permeability, etc., but, as expected, the ODE model fails to report on the fluctuations in the system. The stochastic model predicts that *p*H fluctuations decrease during acidification, but noise analysis of single-vesicle data confirms our finding that the experimental noise is dominated by the fluorescent dye, and it reveals no insight into the true noise in the proton fluctuations. Finally, we again use the reduced DS model explore the acidification of large, lysosome-like vesicles to determine how stochastic elements, such as variations in proton-pump copy number and cycling between on and off states, impact the *p*H setpoint and fluctuations around this setpoint.

## Author summary

Organelles harbor specific ion channels, transporters, and other molecular components that allow them to achieve specific intracellular ionic conditions required for their proper function. How all of these components work together to regulate these concentrations, such as maintaining a specific *p*H value, is complex, and continuum mathematical models have been helpful for evaluating different mechanisms and making quantitative predictions that can be tested experimentally. Nonetheless, organelles can be quite small and some contain only a handful of free protons—can continuum models accurately describe systems with so few molecules? We tested this by creating a discrete, stochastic (DS) model of vesicle acidification that tracks how all of these individual molecules in the vesicle change their state in time. When fitting experimental data, the DS model provides the same parameter estimates as a corresponding continuum model, indicating that both models are equally valid. However, the DS model additionally informs on the noise in the vesicle. When compared to the experimental noise in *p*H, we show that there is no agreement, because experimental fluctuations do not report on the true *p*H fluctuations, but rather they report on the fluctuations in reporter molecule protonation. Given experimental limitations, our result highlights the importance of DS models in predicting noise in organelles.

## Introduction

Acidification of intracellular organelles, such as lysosomes, is achieved by the V-ATPase proton pump. That said, how organelles set and maintain specific *p*H environments remains poorly understood. In addition to H^+^ pumping, counter-ion movement is needed to oppose the buildup of a positive membrane potential [1]. The chloride-proton antiporters and chloride channels of the ClC family are thought to play this role in many organelles [2–5]; however, this role is controversial in the lysosomal where cation channels have been implicated [6]. Another determinant of lumenal *p*H is the proton leak across the membrane, which may be membrane mediated, occur through voltage-gated proton channels such as H_V_1 [7, 8], or through other unknown proteins. Lumenal *p*H is also impacted by internal buffer molecules and the numerous H^+^-dependent chemical reactions that occur in cellular compartments.

Many *p*H regulation studies have focused on assembling a parts list of the components involved in acidification, and in doing so, they have produced valuable information regarding the impact of specific proteins/molecules on *p*H. However, there are still gaps in these lists for most organelles with few studies producing a comprehensive dissection of the protein makeup as was done for the synaptic vesicle by the Jahn lab [9]. Additionally, most cell based studies have reported only ensemble averaged *p*H measurements for different organelles, potentially masking important compartment-to-compartment variations. Exceptions include a handful of studies on endosomes [10], synaptic vesicles [11], and reconstituted proteoliposomes [12]. In particular, the Krishnan lab nicely shows spatial and temporal variation in the distribution of *p*H values of maturing endosomes with final lysosomal values of 5.0 ± 0.16 for 80 individual compartments [10]. Nonetheless, the fluctuations in *p*H of individual compartments in time has not been presented in a cell based assay to our knowledge. In a similar vein, the Grinstein lab showed that lysosomes adjacent to the nucleus are more acidic than those that are distantly positioned [13], and such heterogeneity may have functional consequences related to nutrient response and organelle position [14]. Nonetheless, without knowing how the *p*H in isolated organelles vary we have an incomplete picture of organelle function. For instance, are all lysosomes near *p*H 5, or is there a wide distribution of values in the cell ranging from mildly acidic to very acidic? If this spread is too large, it is possible that some organelles may fail to achieve the chemical environment required for proper function. On a related note, how does the *p*H of an individual organelle change in time as enzymes and pumps activate and deactivate, compartments fuse together changing the chemical make up of the membrane proteins and lumenal contents, and the organelle ages?

Previous models based on ordinary differential equations (ODEs) have proven beneficial in teasing out the contribution of specific components to this rather complex processes. These models include components such as ATP-driven proton pumps, counter-ion fluxes, membrane potential effects, and buffering capacity [15], and they have highlighted the importance of the balance between V-ATPase proton pump and proton leak in setting *p*H along the secretory pathway [16], helped tease apart glutamatergic and GABAergic effects in synaptic vesicles [11], elucidated the role of ClC-7 antiporters in aiding lysosomal acidification [17], and generated hypotheses regarding the complex orchestration of plasma membrane, cytoplasmic, and ruffled border elements during osteoclast-mediated bone resorption [18]. Nonetheless, ODE models only provide an average description of a population of compartments, which may limit their usefulness for studying isolated compartments of a heterogeneous nature. In particular, the continuum approximation may fail for small compartments containing a handful of chemical species. A synaptic vesicle, for example, is typically 20 nm in radius and has to maintain a lumenal *p*H of approximately 5.5 to properly package neurotransmitters. The concentration of free protons at *p*H 5.5 in this small volume however corresponds to only ∼ 0.06 free protons in number. The variables in ODE models can take on fractional values such as these, but non-integer values lack physical meaning.

Recent experiments in the Stamou Lab measured for the first time the acidification of single vesicles by single proton pumps [12]. AHA2, a P-type H^+^ pump from the plant *Arabidopsis thaliana*, was purified and reconstituted into proteoliposomes, and acidification of isolated vesicles was monitored through the *p*H sensitive fluorophore pHrodo. Individual traces showed a great deal of variability both in time and between proteoliposomes, in part because they revealed for the first time that AHA2 stochastically switches between active and inactive states. An ODE model was constructed to fit the time-dependent *p*H traces, and although it provided reasonable parameter estimates and new molecular insight into AHA2-mediated proton leak, the model only describes the mean vesicular *p*H providing no insight into the experimental fluctuations. Moreover, some proteoliposomes were so small that they likely contained less than 1 free proton even at acidic *p*H values. To address these shortcomings and build a framework for answering the questions above, we developed a discrete, stochastic (DS) model of vesicular acidification that enforces the discrete nature of particles while also capturing information about fluctuations in the system. We revisited the AHA2 data, which is all taken from our previous publication [12], using a DS model that is equivalent in complexity to our previous ODE model of acidification and report here that both models provide very similar parameter estimates, even when free proton counts are much less than one. The DS model coupled with analytic results also shows that the noise in these experimental traces is very large and primarily reports on the fluorescent dye rather than the true proton fluctuations. We suggest conditions under which the fluctuations in the reporter dye would more closely match those of the free proton count. Finally, the DS model was used to predict *p*H fluctuations in lysosomal-sized compartments containing different numbers of proton pumps. While the DS model shows that such compartments can achieve different mean *p*H values by modulating the number of pumps recruited to their membrane, it also shows that the distribution of *p*H values around these means are relatively narrow and robust to stochastic activity and Poisson-like changes in pump number.

## Materials and methods

### A discrete, stochastic model of acidification

**Fig 1:**
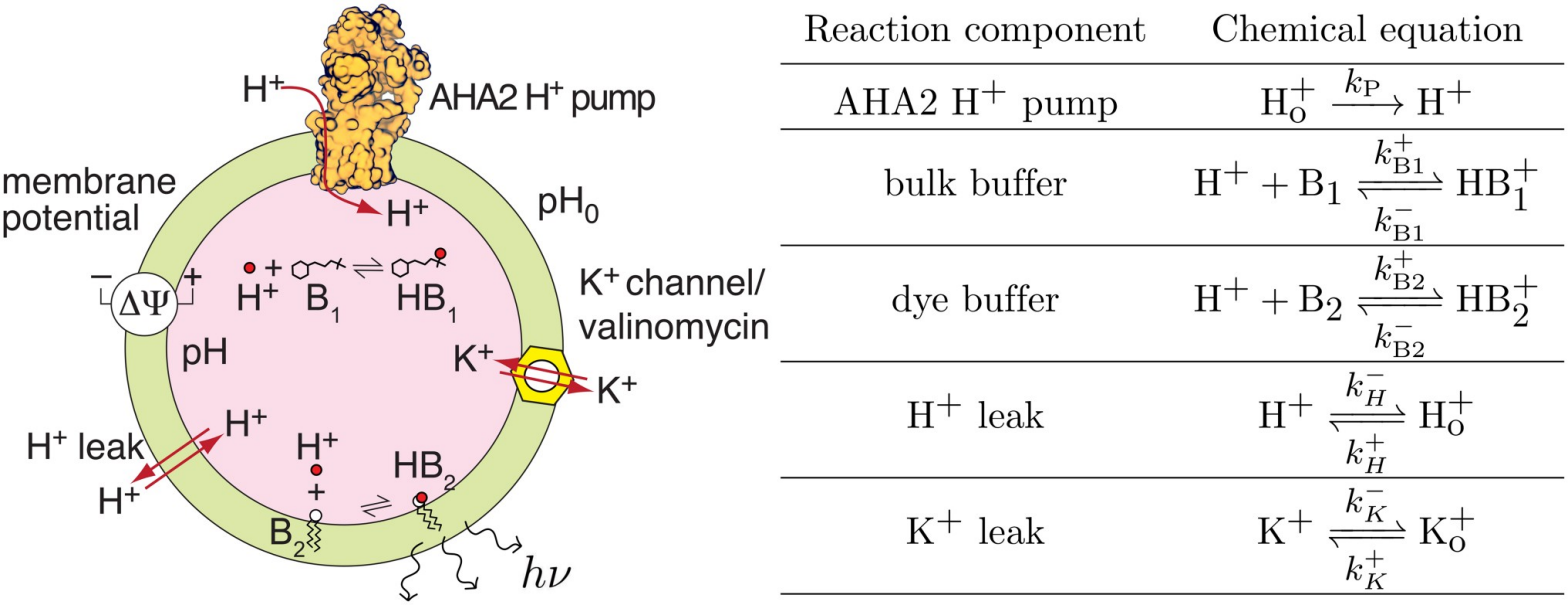
Schematic representation of vesicular acidification model. **(A)** Cartoon representation of the model with all elements and reactions explicitly shown. **(B)** Chemical equations and rate constants for each model component.

We model the acidification of single vesicles using a system of chemical reactions following our previous ODE work [12]. These reactions account for proton and potassium leaks, a proton pump, lumenal buffer species, and impermeant, negative Donnan particles trapped in the lumen (Fig 1). These reactions determine the change in lumenal concentrations and the membrane potential, and the corresponding ODE for the number of lumenal H^+^, for example, is given by:

$$\begin{array}{c} \frac{d}{dt}N_{H}=\underset{\text{pump}}{\underset{︸}{k_{P}}}+\underset{\text{leak}}{\underset{︸}{N_{A}\left(k_{H}^{+}{\left[H^{+}\right]}_{o}-k_{H}^{-}\left[H^{+}\right]\right)}}+\underset{\text{buffer}\;\text{reactions}}{\underset{︸}{N_{A}V\left(k_{B}^{-}\left[HB^{+}\right]-k_{B}^{+}\left[B\right]\left[H^{+}\right]\right)}}, \end{array}$$ (1)

where *k*_P_ is the time-dependent proton pumping rate into the vesicle, *V* is the volume of the vesicle (assumed spherical throughout), $k_{H}^{+}$ and $k_{H}^{-}$ are rate constants describing the passive movement of protons into and out of the vesicle, respectively, $k_{B}^{-}$ and $k_{B}^{+}$ are rate constants for the dissociation and association of the buffer and proton, respectively, [*HB*^+^] and [*B*] are the concentrations of the protonated and free buffer, respectively, [*H*^+^]_o_ and [*H*^+^] are the concentrations of protons outside and inside the vesicle, respectively, and $N_{A}$ is Avagadro’s number. Throughout, the subscript o refers to an extracellular value and lumenal values do not have a subscript. Many of these quantities are subject to further biophysical and chemical constraints.

#### Buffer reactions

While Eq 1 only shows a single species, our model has two explicit buffer molecules: solution buffer molecules distributed throughout the vesicle, *B*_1_, and lipid-bound dye molecules called pHrodo, *B*_2_. In general, detailed balance relates the forward and reverse rates through the experimentally determined *p*K_*a*_ value for each buffer (see Table 1) as follows:

$$\begin{array}{c} k_{B}^{+}=10^{pK_{a}}\cdot k_{B}^{-} \end{array}$$ (2)

For all simulations, the value of $k_{B}^{-}$ was $0.1\frac{1}{s}$ for both buffer species, and the forward rates then followed from Eq 2 and are given in Table 1. The value of the reverse rate was selected such that the forward buffer rates (∼ 10^4^-10^5^ $\frac{L}{mol\cdot s}$) would be the fastest in the simulation. The change in protonated, *N*_*HB*_, and free buffer molecules, *N*_*B*_, for either species are then given by:

$$\begin{array}{c} \frac{d}{dt}N_{HB}=N_{A}V\left(k_{B}^{+}\left[B\right]\left[H^{+}\right]-k_{B}^{-}\left[HB^{+}\right]\right) \end{array}$$ (3)

$$\begin{array}{c} \frac{d}{dt}N_{B}=N_{A}V\left(k_{B}^{-}\left[HB^{+}\right]-k_{B}^{+}\left[B\right]\left[H^{+}\right]\right), \end{array}$$ (4)

where the constraint on the total number of buffer molecules, *N*_*HB*_ + *N*_*B*_ = *N*_*T*_, eliminates one of the equations. [*B*_1_] is constant in all experiments, and *N*_*T*_ = *V*[*B*_1_]. pHrodo lipid-dye molecules are incorporated into vesicles at a mole fraction F of 1.5 to 1000, and then the vesicle geometry defines the average number of molecules and concentration:

$$\begin{array}{c} \left[B_{2}\right]=\frac{N_{T}}{V}=\frac{1}{V}\cdot \underset{N_{T}}{\underset{︸}{\left(\frac{A}{A_{L}}\cdot \frac{F}{N_{A}}\right)}}, \end{array}$$ (5)

where $A_{L}$ is the area per lipid headgroup, and *A* is the vesicle surface area.

**Table 1:** Model parameters.

description	symbol	value	units
liposome radius	*r*	variable	nm
liposome area	*A*	variable	nm^2^
liposome volume	*V*	variable	nm^3^
AHA2 pumping rate	*I*_*P*_	variable	H^+^/s
membrane H^+^ permeability	*P*_*H*_	variable	cm/s
AHA2 H^+^ permeability	*P*_AHA2_	variable	cm/s
external potassium permeability	*P*_*K*_	1.1 × 10^−7^	cm/s
external potassium concentration	[*K*^+^]	100	mM
bulk buffer concentration	[*B*_1_]	10	mM
bulk buffer *p*K_a_	*p*K_a_^1^	6.1	–
bulk buffer forward rate	$k_{B1}^{+}$	1.3 x 10^5^	$\frac{L}{mol\cdot s}$
pHrodo concentration	[*B*_2_]	see Eq 5	mM
pHrodo *p*K_a_	*p*K_a_^2^	5.72	–
pHrodo forward rate	$k_{B2}^{+}$	5.2 x 10^4^	$\frac{L}{mol\cdot s}$
area per lipid head group	$A_{L}$	0.7	nm^2^
mole fraction pHrodo-to-lipid	F	1.5/1000	–
Faraday’s constant	F	96,485	C/mol
gas constant	R	8.31	J/(K⋅ mol)
temperature	T	293	K
membrane capacitance	C	10^−6^	F/cm^2^

Model parameter values were set to match the experimental parameters of the Stamou Lab’s single vesicle recordings [12].

#### Membrane potential

We employ a physical model for the membrane potential [19] determined by summing the total charge in the vesicle and dividing by the membrane capacitance *C*:

$$\begin{array}{c} \Delta \psi =\frac{F}{N_{A}AC}\left(N_{H}+N_{{HB}_{1}}+N_{{HB}_{2}}+N_{K}-N_{A}VB\right), \end{array}$$ (6)

where *N* indicates numbers of specific charged molecules (free protons, protonated buffer, protonated dye molecules, and potassium, respectively), *B* is the concentration of negatively charged Donnan particles, and *F* is Faraday’s constant (see Table 1 for values).

#### Passive H^+^ & K^+^ permeability

Passive H^+^ and K^+^ membrane leaks were modeled as:

$$\begin{array}{c} \frac{d}{dt}N_{K}=N_{A}\left(k_{K}^{+}{\left[K^{+}\right]}_{o}-k_{K}^{-}\left[K^{+}\right]\right), \end{array}$$ (7)

where this equation for K^+^ is equivalent to the leak term in Eq 1 with similar definitions, and the rate constants $k_{X}^{+}$/$k_{X}^{-}$ for *X* = *K* or *H* depend on Δ*ψ*. In the absence of a membrane potential, the flux vanishes when the internal and external concentrations are equal leading to $k_{X}^{+}\left(\Delta \psi =0\right)=k_{X}^{-}\left(\Delta \psi =0\right)=k_{X}^{0}$. In the presence of a potential, the base rates must be modified to obey the Nernst equation and provide the correct equilibrium condition. The simplest model for a monotonic cation that enforces reversibility is the following:

$$\begin{array}{c} \frac{k_{X}^{-}}{k_{X}^{+}}=e^{\frac{\Delta \psi F}{RT}}, \end{array}$$ (8)

where *R* is the gas constant and *T* is temperature. We chose to split the membrane potential dependence equally among the forward and reverse reaction rates:

$$\begin{array}{l} \begin{array}{c} k_{X}^{-}\left(\Delta \psi \right)=k_{X}^{0}e^{+\frac{\Delta \psi F}{2RT}} \end{array}\\ \begin{array}{c} k_{X}^{+}\left(\Delta \psi \right)=k_{X}^{0}e^{+\frac{\Delta \psi F}{2RT}} \end{array} \end{array}$$ (9)

The membrane permeability (cm/s) is related to A (nm^2^) and the reaction constant, $k_{X}^{0}$ (L/s), via the equation:

$$\begin{array}{c} P_{X}=\frac{k_{X}^{0}}{A}\cdot 10^{17}. \end{array}$$ (10)

$k_{K}^{0}$ for K^+^ is zero prior to valinomycin addition and afterwards is set by Eq 10 and its membrane permeability (Table 1). $k_{H}^{0}$ for H^+^ is composed of two terms:

$$\begin{array}{c} k_{H}^{0}=k_{\text{lipid}}^{0}+k_{\text{AHA}2}^{0}. \end{array}$$ (11)

The first term is direct membrane mediated leak that is always present, and the second term is proton pump dependent leaking that occurs once the pump stochastically turns off as described previously [12]. The model parameters in Table 1, *P*_*H*_ and *P*_AHA2_, refer to the membrane permeability calculated from $k_{\text{lipid}}^{0}$ and $k_{\text{AHA}2}^{0}$ individually using Eq 10.

#### Implementation of the stochastic model

The ODE-based model of vesicular acidification summarized by Eqs 1, 4, and 7 was implemented in the biochemical reaction network software, COPASI [20], using the time course feature. Only the internal concentrations were allowed to vary, and the model held all extracellular concentrations fixed. To simulate a discrete, stochastic (DS) process, COPASI employs a version of the classic Gillespie algorithm called the Next Reaction Method [21]. The reaction equations, rate constants, and starting concentrations from Fig 1 were entered into COPASI, and a trajectory of reactions and corresponding reaction times were stochastically generated. After each reaction was executed, the the membrane potential was updated according to Eq 6 as well as all affected rate constants. Each simulation was run for 750 s, and species counts were recorded every 0.02 seconds. For comparisons to deterministic ODE results, we simultaneously solved Eqs 1, 4, and 7 using the ode45 stiff solver algorithm in MATLAB (The MathWorks, Natick, MA).

#### Calculating *p*H

We calculated lumenal *p*H from the stochastic vesicle model results using three methods. First, post-simulation, the instantaneous free proton count at each time step was converted to a *p*H value based on the volume of the vesicle using the relation *p*H = −log_10_([H^+^]), which we simply refer to as *p*H. Second, we time-averaged the proton count over a time Δ*t*, usually 0.5 s, and then took the $-{\text{log}}_{10}\left(\left[\bar{H^{+}}\right]\right)$ to determine the time averaged $\bar{pH}$ from the DS model. Third, we computed *p*H from the fluorescence of the pHrodo dye molecules to estimate *p*H in a manner identical to previously published experiments [12], *p*H^*hν*^. When pHrodo is protonated, it fluoresces, and a CCD camera detects the number of photons emitted in time windows from isolated vesicles. The *p*H was then determined from the photon count using a predetermined vesicle-size specific calibration curve.

We first solved the stochastic model to determine the number of protonated dye molecules in time. Next, we estimated the average photons emitted from *N*_*HB*_ dye molecules using the equation:

$$\begin{array}{c} {\bar{N}}_{h\nu }=c\cdot N_{{HB}_{2}}+b \end{array}$$ (12)

where *c* and *b* are trace-dependent constants described later. Realizing that emission is stochastic, we simulated the actual number emitted photons assuming they obey Poisson statistics:

$$\begin{array}{c} N_{h\nu }\left(t\right)=P\left({\bar{N}}_{h\nu }\right)=P\left(c\cdot N_{{HB}_{2}}\left(t\right)+b\right), \end{array}$$ (13)

where *P* is the Poisson distribution with mean ${\bar{N}}_{h\nu }$. We determined *c* and *b* for each experimental trace by using the same linear model to convert protonated dye molecules to photon count. To solve for the two unknown parameters, we used the average experimental *p*H (*p*H_*exp*_) and *N*_*hν*_ values prior to ATP addition (*T*1) and after maximum acidification was reached (*T*2) to obtain two constraint equations:

$$\begin{array}{c} \begin{array}{c} N_{h\nu }^{T1}=c\cdot N_{{HB}_{2}}^{T1}+b\\ N_{h\nu }^{T2}=c\cdot N_{{HB}_{2}}^{T2}+b, \end{array} \end{array}$$ (14)

where $N_{{HB}_{2}}$ was obtained from *p*H_*exp*_ using the Henderson-Hasselbach equation:

$$\begin{array}{c} N_{{HB}_{2}}=\frac{N_{T}}{1+10^{\left(pH_{exp}-pK_{a}\right)}}, \end{array}$$ (15)

where *N*_*T*_ is the total number of dye molecules. After solving for *c* and *b*, the stochastic model reproduced the experimental photon count time series very well using Eq 13. Finally, the model *p*H in this third method is determined as follows:

$$\begin{array}{c} pH^{h\nu }=pK_{a}^{2}+\log_{10}(\frac{N_{T}-N_{{HB}_{2}}}{N_{{HB}_{2}}}). \end{array}$$ (16)

where importantly $N_{{HB}_{2}}$ is not determined here from the model, but rather from Eq 12 as $N_{{HB}_{2}}=N_{h\nu }/c-b$, which includes photon shot noise. Our modeling efforts not only match the photon time series, but it also closely matches the experimental *p*H calculation curves in Ref. [12].

#### Data fitting

We used our models to fit experimental *p*H measurements using the Nelder-Mead search algorithm [22] to find the optimum parameter values that minimized the root mean squared deviation (RMSD) between the simulated and the experimental data across the entire time series:

$$\begin{array}{c} RMSD=\sqrt{\sum_{i=1}^{N}(X_{mod}\left(t_{i}\right)-X_{exp}\left(t_{i}\right){)}^{2}}, \end{array}$$ (17)

where *N* is the number of experimental data points and *X*_*mod*/*exp*_ is the model/experimental free proton count or *p*H, as described next. When computing *p*H directly from the free proton count in the DS model, we compared calculated free proton values to the experimental free proton count, which was back calculated from the experimental *p*H values. However, when we computed *p*H from the photon count in the DS model, we compared *p*H^*hν*^ directly to the experimental *p*H using Eq 17. Finally for the ODE model, computed *p*H was again compared directly to the experimental *p*H. We chose to use free proton count in the first case, because *p*H is undefined when there are no free protons. Because the RMSD value for any given parameter choice varied from run-to-run, due to stochastic variation, we carried out the optimization on the average RMSD from 10 runs: $err=1/10\sum_{j=1}^{10}RMSD_{j}$. Optimum fits were determined by varying *k*_P_, $k_{\text{lipid}}^{0}$, $k_{\text{AHA}2}^{0}$, relating to the pumping rate, passive membrane permeability, and AHA2-dependent proton leak. All other parameters listed in Table 1 were fixed. The initial interior proton concentration was set to the experimental value and was also assumed to be in equilibrium with the exterior *p*H, because ATP had not yet been added. The ratios of protonated to deprotonated buffers were set based on the total buffer concentrations and known buffer *p*K_*a*_ values. Additionally, the radius, *r*, of each vesicle was measured in previous experiments [12] wherein individual radii were determined by converting diffraction-limited intensity spots to physical proteoliposome size [23]. These vesicle sizes were then calibrated via cryo-EM [12, 23].

Experimentally, AHA2 H^+^ pumping (*I*_*p*_) begins with the addition of ATP to solution and then stochastically halts at some later time. We accordingly divided each trace into three sections: before, during, and after pumping. *I*_*p*_ was non-zero only in the middle section, and *P*_AHA2_ was only non-zero after pumping due to AHA2.

## Results

### The model reveals discrete *p*H jumps not observed in experiment

We first used the stochastic model to explore the acidification of a small vesicle ∼ 50 nm in radius. While still larger than synaptic vesicles, such a small space (5 × 10^−7^ pL) poses an interesting question when interpreting proton concentrations since a *p*H of 5 corresponds to ∼ 3 free protons. To our knowledge, our recent work, led by the Stamou lab, represents the only experimental study of the acidification of isolated vesicles by single transporters, which we call Single Transporter Activity Recordings (STARs). These artificial proteoliposomes reconstituted with 1 or a few AHA2 proton pumps, verified with single molecule quenching of tagged AHA2 [12], ranged in size from tens of nanometers to hundreds of nanometers, and they serve as an excellent standard to compare against our discrete, stochastic model.

**Fig 2:**
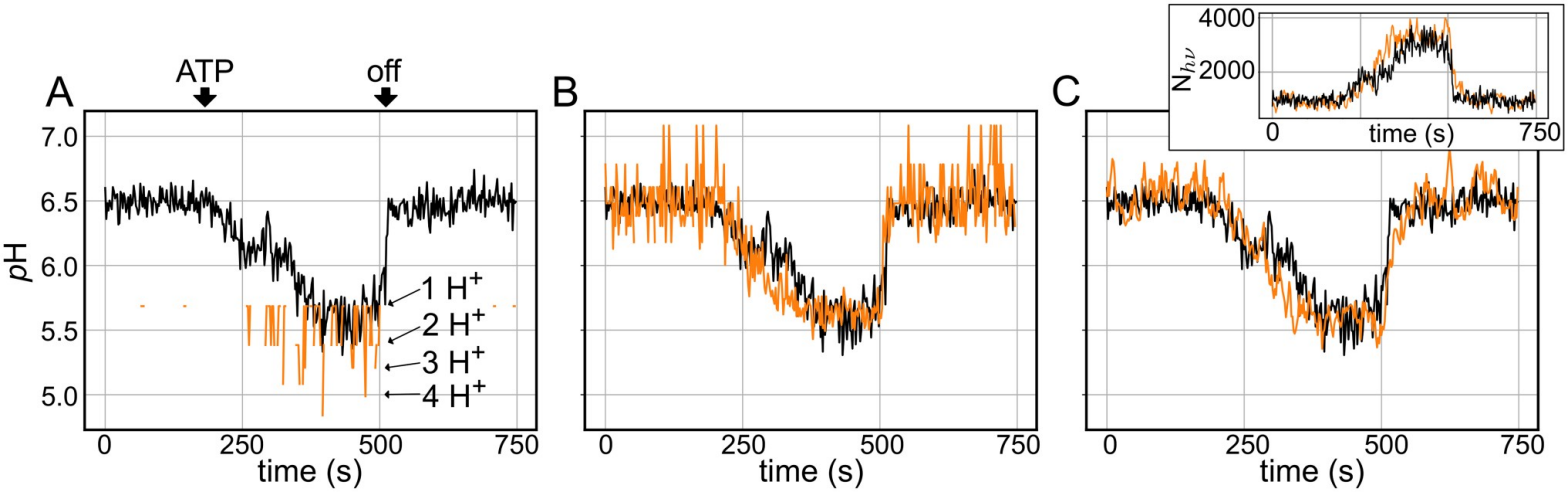
Discrete, stochastic simulations of a small vesicle. **(A)** The instantaneous *p*H from the DS model simulation (orange) plotted over an experimental trace obtained from a 58 nm radius vesicle (black). The same experimental trace is shown in all three panels, and the times when ATP are added and the pump stops are indicated with arrows. **(B)** Time averaged DS model results using a 0.5 s window to produce $\bar{pH}$ (orange). **(C)** *p*H determined from fluorescent reporter (*p*H^*hν*^) calculated from the modeled photon count emitted from the pHrodo dye (orange). The inset shows the corresponding experimental (black) and simulated (orange) photon count, *N*_*hν*_, over time.

Fig 2 shows the time resolved trace from a 58 nm vesicle (black curve in all panels). Near 180 s, ATP is added to solution to initiate pumping of the AHA2 proton pump, at which point the vesicle acidifies from the bathing *p*H of 6.5 to 5.5. Then near 500 s this transporter spontaneously turns off and protons leak out of the vesicle returning the intravesicular *p*H to the exterior value. We first carried out curve fitting to obtain a set of model parameters that most closely matches the experiment following the optimization scheme outlined in the Methods. The orange curve in Fig 2A is a representative stochastic run of the DS model using these optimized parameters. In this simulation, we are using the instantaneous number of free protons in the vesicle at each point in time and then converting that value to *p*H using the standard definition of *p*H (orange traces). It is immediately clear that the model produces a result that is dramatically different from the experimental trace. As expected, the model shows discrete changes in the state of the system when 0, 1, 2, 3, or 4 free protons are present in the lumen—*p*H 5.7 corresponds to 1 proton, 5.4 to 2, and so on. For a large portion of the beginning and end of the simulation trace, the instantaneous proton count is zero, resulting in an infinite *p*H value not visualized in Fig 2A. Despite the clear differences, there are similarities between the simulation and experiment. When AHA2 starts pumping, there is a general acidification that occurs in both traces, and when pumping stops both alkalinize. While the vesicle returns to the extravesicular *p*H of 6.5, the DS model returns to having no protons on average (*p*H → ∞). While we suspect that the DS model is an excellent representation of the true biological system, and that it likely produces the correct instantaneous *p*H values, why does it not match experiment?

In a first attempt to address this question, we accounted for the fact that the experiment does not report on the instantaneous *p*H. The fluorescence emitted from acidifying vesicles was collected over a 0.5 second window and reported every 2 seconds [12]. Thus, we time averaged the free proton count from the DS model over a 0.5 second window prior to converting to *p*H ($\bar{pH}$). As can be seen in Fig 2B, this approach produces a continuous *p*H trace that no longer shows discrete steps in proton count and more closely matches experiment. The alkaline *p*H values correspond to fractional numbers of free protons, and they result from many snapshots of the vesicle with no protons and a few with one. Although this method of time averaging produced a simulated *p*H curve with the same mean *p*H values as the experimental trace, there are pronounced differences in the magnitude of *p*H fluctuations between the two curves. In the model, the fluctuations are quite large at alkaline *p*H levels and are suppressed as the vesicle’s mean lumenal *p*H decreases. This trend is simply a consequence of the log scale of *p*H measurement (i.e. while variance in the number of free protons grows at acidic *p*H values, the log_10_ suppresses the fluctuations); however, it was puzzling that the experimental traces showed no obvious *p*H dependent change in the magnitude of the fluctuations, as can be seen in the example curve in Fig 2.

We also modeled the *p*H in the vesicle in a manner that exactly mimics the experimental setup to gain deeper insight into what the experiments are telling us. As is the case with many environmental reports, the fluorescence is not a direct readout of the free molecule/ion of interest, but rather it is the result of binding of that molecule/ion to a reporter, which then changes its fluorescent properties. Here, the reporter is the *p*H sensitive dye, pHrodo, which resides in the membrane leaflet due to conjugation to a DOPE lipid. For a 58 nm radius vesicle, there are approximately 88 pHrodo lipids in the inner leaflet. Given the *p*K_*a*_ of 5.72 of this lipidated form of pHrodo (see Ref. [12]), on average, initially 12 lipids are protonated, and this number increases to 51 as the system acidifies. As described in the Methods, we simulated the stochastic emission of photons from each bound pHrodo, which also increases in time (Fig 2C inset), and we plotted the predicted lumenal *p*H from this signal in the main panel, which we call *p*H^*hν*^. *p*H^*hν*^ not only tracks the mean experimental *p*H, but also qualitatively the experimental fluctuations. The two approaches for interpreting the DS model exhibit very different fluctuations because one is based on proton count (panel B) and one is essentially based on bound dye count (panel C), and both quantities and their variances scale very differently with *p*H. Proton count changes exponentially with *p*H, while the bound dye count, and hence the photon count, increases almost linearly over the experimental range of interest (Fig 2C inset). Although the *p*H^*hν*^ best fits the experimental results, it highlights the fact that the experimental measurements are not directly capturing information about the free protons, but rather the fraction of protonated pHrodo molecules. Hence, we assert that the instantaneous *p*H calculations determined directly from the DS model provide the best indication of the free proton dynamics in these compartments.

### Parameter estimates are model independent

**Fig 3:**
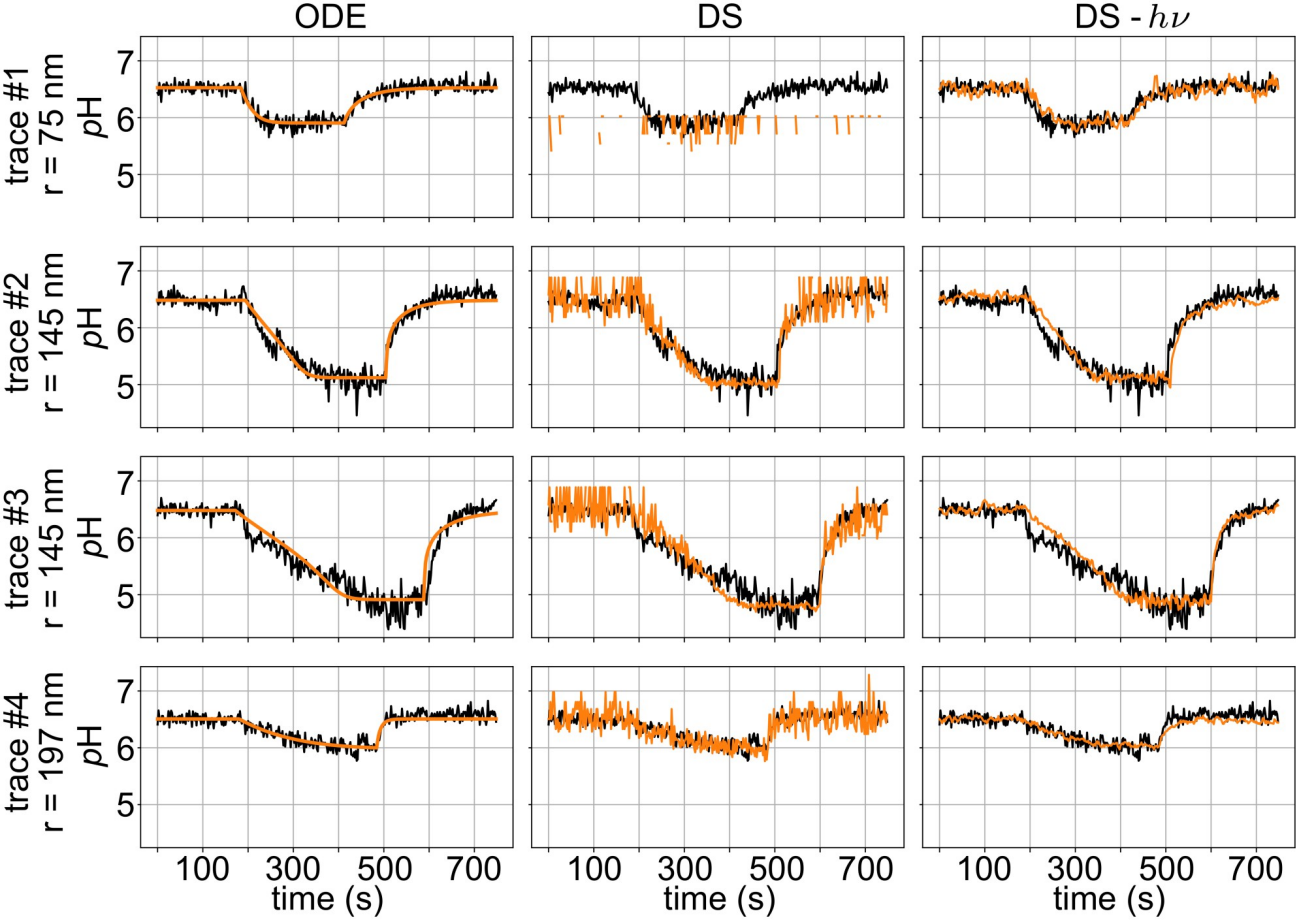
Liposome acidification dynamics using the ODE and DS models. ODE model fits along with representative simulations from the DS model with *p*H interpreted from free proton counts or photon counts (indicated by *hν*) for the four experimental traces in Table 2. Each row is a different experimental trace plotted in black, and all simulations are orange. The first, second, and third columns contain the ODE, DS model, and DS model using photon count results, respectively. In the last column, the calculated *p*H is *p*H^*hν*^. All parameter values can be found in Table 2.

Next, we wanted to apply our two methods of fitting the stochastic model to a range of STAR data recorded from vesicles of different sizes, quantitatively compare the parameter estimates from each approach, and importantly, compare the results to solutions derived from ODE calculations. The vesicles in Fig 3 range from 75 to 197 nm in radius (a volume difference of approximately 18×), and for each trace, we fit the DS and ODE models to the data and identified the optimal AHA2 H^+^ pump rate (*I*_*P*_), membrane proton permeability (*P*_*H*_), and the AHA2-dependent proton permeability (*P*_AHA2_) that occurs after the pump stops (Table 2). Since the DS model is stochastic, any individual simulation may provide a poor match to a given STAR even if the parameter values are optimal. Therefore, for each point in parameter space, we ran the stochastic models 10 times and averaged the goodness of fit from each run to provide a representative score, which was then optimized using a Nelder-Mead search algorithm [24].

**Table 2:** Parameter estimates for the DS and ODE models.

trace	r (nm)	method	*I*_*P*_ (H^+^/s)	*P*_*H*_ (cm/s)	*P*_AHA2_ (cm/s)
1	75	DS	117	31 × 10^-5^	8.5 × 10^-5^
		DS—*hν*	122	29 × 10^-5^	10 × 10^-5^
		ODE	119	28 × 10^-5^	7.1 × 10^-5^
2	145	DS	471	3.0 × 10^-5^	140 × 10^-5^
		DS—*hν*	482	4.0 × 10^-5^	66 × 10^-5^
		ODE	448	3.9 × 10^-5^	65 × 10^-5^
3	145	DS	286	1.1 × 10^-5^	39 × 10^-5^
		DS—*hν*	280	1.3 × 10^-5^	153 × 10^-5^
		ODE	252	1.3 × 10^-5^	46 × 10^-5^
4	197	DS	475	19 × 10^-5^	521 × 10^-5^
		DS—*hν*	436	20 × 10^-5^	648 × 10^-5^
		ODE	425	19 × 10^-5^	514 × 10^-5^

Model fits (orange curves) are reported in separate columns superposed on the corresponding experimental trace (black). The ODE model (Fig 3 left column) produces piece-wise smooth curves that overlay well with the experimental data, throughout the trace; however, as discussed earlier, the ODE model does not reproduce the fluctuations in the trace. The greatest deviations between the fit and the data occur in traces 2 and 3 around 150-175 seconds. At these points in time, the experimental trace changes rapidly, either due to a stochastic fluctuation or to the pump rate changing in a manner not encoded in the model. For the DS model (middle column), *p*H produces discrete *p*H jumps, as discussed earlier, not observed in the data recorded from the small 75 nm radius vesicle (trace 1), but the discrete nature is obscured in the larger vesicles (traces 2-4) because they contain many more protons. Incidentally, the ODE model predicts a free proton concentration less than 1 for most of trace 1. As the vesicle acidifies in the DS method for traces 2-4, the noise decreases for reasons discussed previously, and this is not observed in the data. Lastly, interpreting *p*H changes in the DS model by explicitly modeling the emitted photons (right column) produces the closest fit to the experimental data for the small vesicle (trace 1), and as discussed before, it exhibits similar noise at both high and low *p*H. However, the magnitude of the fluctuations for the larger vesicles is smaller than what is observed experimentally, which may arise from other environmental sources not included in our model. As we show in Fig A in S1 File, the membrane potential is predicted to be negligible for the DS model due to sufficient clamping by potassium via valinomycin.

Importantly, despite qualitative differences in the ODE and DS model, they both predict very similar parameter estimates (Table 2). Both DS methods predicted pump values (*I*_*P*_) within 10% of the ODE value, while the passive membrane permeability values (*P*_*H*_) are all between 0 and 30%. The ODE results for *P*_*H*_ match the DS model interpreted from photon counts better, but we are unsure if this is true or the result of analyzing a small number of traces. The greatest variability for the DS method predictions is in the estimated *P*_AHA2_ values, which differ for the two DS methods by a factor of 2-4 for traces 2 and 3. We note that estimates of this quantity, which represents the leak of protons through the transporter once it stops pumping, are prone to error. Since the protons leave the vesicle so quickly over a short period of time, as can be seen near 500 s in Fig 2, the value is poorly constrained from above. That is, increasing *P*_AHA2_ above a critical value produces nearly identical looking fits with similar RMSD values. Thus, we do not think that these differences are crucial. Rather, the closeness in all predicted values across all models and methods suggests that it is not essential to model the fluorescent response of the dye molecules to capture the mean behavior of the system nor does one need to model the discrete nature of the proton movement, as we elaborate upon next.

**Fig 4:**
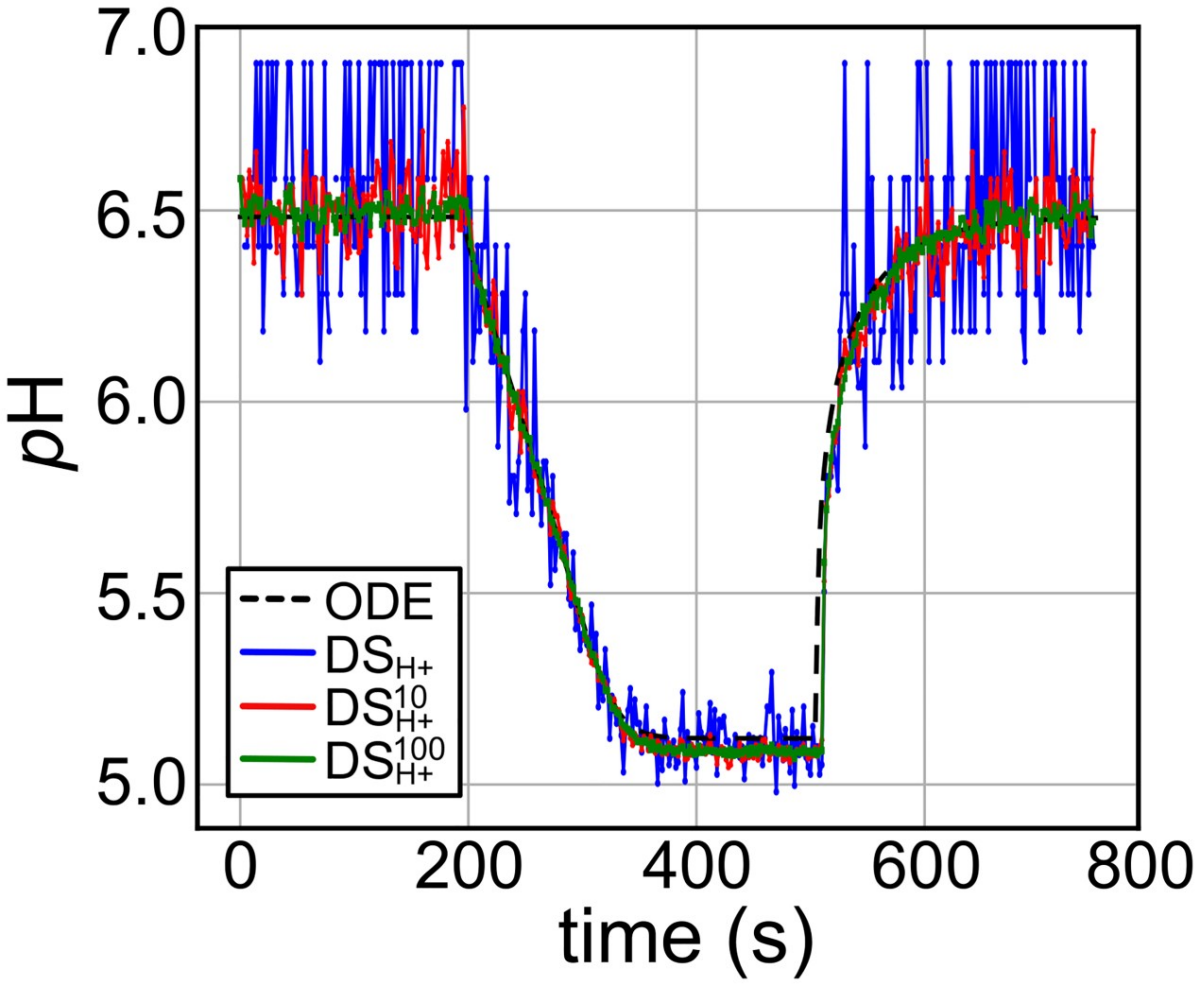
ODE model fit is average of many DS simulations. The ODE and DS model fits for Trace 2 from Table 2, *r* = 145 nm, is displayed. An average of 10 and 100 DS simulations with the DS method predicted parameter values is displayed as well. As the number of averaged DS simulation increases, the ODE model fit is approached.

One reason why the mean parameter estimates for the ODE and DS models are close is because the ODE model can be interpreted as an average of multiple DS model simulations, as we show in Fig 4. Here, we used the parameter estimates for trace 2 and ran the DS model one time (blue), averaged over 10 runs (red), or averaged over 100 runs (green) and plot the resulting *p*H. As more traces are averaged, the fluctuations are suppressed, and the final curve is indistinguishable from the smooth trace produced from the ODE model (black dash). Evidently, the ODE model accurately describes the mean lumenal *p*H of the liposome over time. The similarity between ODE and DS model predictions suggests that the deterministic, continuum model is detailed enough to provide parameter estimates, even for very small vesicles with large *p*H fluctuations, and proton counts that are at times less than one.

Finally, we carried out sensitivity analysis on the ODE model for trace 2 to determine which parameters most greatly impact the dynamics and steady state behavior. Given the similarity between mean parameter estimates for both DS and ODE models, we expect that these results apply equally to both. As with several of our previous *p*H regulation studies, the pumping rate, proton permeability, and surface area all impact the steady state *p*H the greatest, while the properties of the primary buffer, AHA2 pumping rate, and volume impact the rate of acidification (Table A in S1 File).

### Vesicle radius and mean *p*H are the greatest determinants of *p*H fluctuations

**Fig 5:**
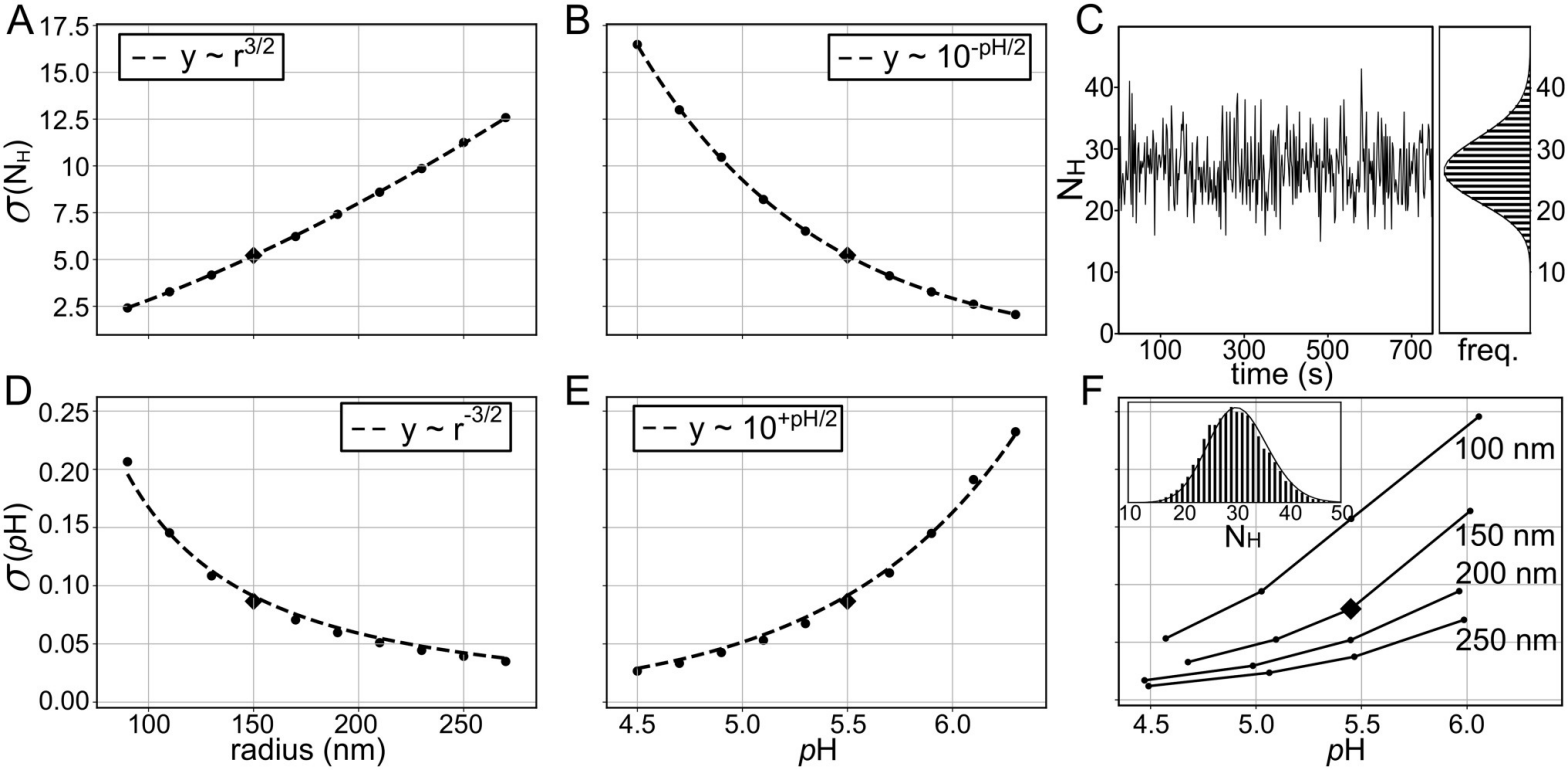
Effects of vesicle radius and steady state *p*H on H^+^ and *p*H fluctuations as predicted from our DS model. **(A&B)** The standard deviation in the free proton count of DS model simulations (*σ*(N_H_)) as a function of vesicle radius with the lumenal/bath *p*H set to 5.5 (panel A) or as as a function of lumenal/bath *p*H with the radius fixed at 150 nm (panel B). **(C)** The instantaneous free proton count over time and corresponding count histogram for the common DS model simulation to panels A-D (diamonds) with *r* = 150 nm and *p*H = 5.5. **(D&E)** The standard deviation in *p*H of DS model simulations (*σ*(*p*H)) as a function of vesicle radius with the lumenal/bath *p*H set to 5.5 (panel D) or as as a function of lumenal/bath *p*H with the radius fixed at 150 nm (panel E). Dashed lines in A, B, D, and E scale as expected based on free proton count and *p*H scaling with volume and *p*H as described in the main text. **(F)** Standard deviation in steady state *p*H as a function of lumenal *p*H for vesicles undergoing active proton pumping. The proton pump rate was adjusted to achieve different internal *p*H values, each curve corresponds to a different size vesicle (*r* = 100, 150, 200, and 250 nm). The inset is a histogram of free proton counts for the *r* = 150 nm, *p*H = 5.45 data point (diamond). For all panels, the simulations of the DS model were run for 750 s at equilibrium with the bath *p*H, with no active pumping (except panel F), and *P*_*H*_ = 4.6 × 10^−5^ cm/s. All other parameters can be found in Table 1.

A central question in intracellular *p*H regulation is how the *p*H setpoint is established in an organelle. A related question is, once set, are vesicles prone to large *p*H fluctuations that would change the proton motive force (PMF) across the membrane influencing H^+^-dependent transporters, ion channels, and lumenal *p*H-dependent enzymes? Since the DS model captures fluctuations, we wanted to address these points computationally by identifying the parameters that most greatly influence the fluctuations in the free proton count, and correspondingly the *p*H. Our analysis revealed that vesicle radius and mean lumenal *p*H exert the greatest impact on the number of total free protons in the system, and hence the H^+^ fluctuations. In Fig 5A, we carried out a series of calculations in which we varied the radius of an idealized liposome from 70 to 270 nm with the external *p*H constant at 5.5. There were no proton pumps, only a passive H^+^ permeability, and the mean lumenal *p*H mirrors the external value for all sizes. We see as the radius of the vesicle increases the standard deviation of free proton count, *σ*(N_H_), also increases (Fig 5A). Next, we kept the vesicle radius constant at 150 nm, but varied the bath *p*H from 4.5 to 6.3, and as the lumen acidifies *σ*(N_H_) increases (Fig 5B). If the free proton count obeys Poisson statistics, we would expect the standard deviation to scale like the square root of the number of protons. For panel A, as the vesicle radius increases at fixed *p*H, the number of free protons is proportional to the vesicle volume, and we would expect $\sigma ({\text{N}}_{\text{H}})∼\sqrt{r^{3}}=r^{\frac{3}{2}}$, as it does (dashed curve). Meanwhile, the vesicle size is fixed as the *p*H changes so the number of free protons scales like 10^−*pH*^, and the noise scales like $\sqrt{10^{-pH}}$ as expected (dashed curve in Fig 5B). In general, the theory is an excellent match to the numerical results (points), supporting the claim that the H^+^ fluctuations obey Poisson statistics at steady state in our model. In panel Fig 5C, this correspondence is explicitly shown for a single dynamic trace of H^+^ from the DS model at *p*H 5.5 and *r* = 150 nm (diamond in Fig 5A and 5B). Binning the counts on the right reveals a histogram with values from 0 to 8 that is again well fit by a Poisson distribution (solid line).

Experimentally, it is more useful to examine the fluctuations in *p*H units than proton count, which we see scales very differently with radius and mean *p*H in panels Fig 5D and 5E, respectively. Standard deviation in *p*H decreases as vesicle radius increases, and it increases as mean *p*H increases. These trends are captured by the dashed curves in panels D and E, which follow from the transformation to *p*H:

$$\begin{array}{c} \sigma \left(pH\right)=\sigma \left(N_{H}\right)\cdot |\frac{\partial pH}{\partial N_{H}}|=\sigma \left(N_{H}\right)\cdot |\frac{-1}{\text{ln}\left(10\right)\cdot N_{H}}|\propto \{\begin{array}{c} r^{-\frac{3}{2}}\\ 10^{+pH/2} \end{array}, \end{array}$$ (18)

where we note that N_H_ scales like *r*^3^ or 10^−*p*H^, and we recall how *σ*(N_H_) varies with *r* and *p*H from the last paragraph. Thus, fluctuations in *p*H are suppressed in larger, more acidic vesicles—the opposite of what is observed for proton count. Finally, we wanted to determine if active pumping changed the Poisson-like characteristic of the noise, so we carried out fluctuation analysis at steady state for an acidic vesicle with active pumping. For different sized vesicles the proton pump rate was varied to achieve different steady state *p*H values, and the resulting standard deviation of *p*H was recorded (panel F). *σ*(*p*H) matches the values in panels D and E (diamonds), suggesting that the process of proton pumping—in our simple model—does not influence *p*H fluctuations. Furthermore, we explicitly show for one of the DS simulations with pumping that at the acidified steady state *p*H, the distribution of free proton counts is still well fit by a Poisson distribution (panel F inset).

### Fluorescent reporters cannot reveal the true *p*H fluctuations in a compartment

Important information regarding the nature of complex physical systems can often be extracted from the natural fluctuations. However, the proton noise analysis presented in Fig 5 is based on a very simple model, and we directly analyzed the noise in the true proton count, which cannot be done experimentally. If real *p*H regulatory systems harbor more interesting feed back systems that deviate from Poissonian statistics, we would have to extract this from a fluorescent reporter, as is often done in single molecule experiments. Thus, we wanted to determine if the true fluctuations in the free proton count could be related to the fluctuations in the fluorescent reporter. The pH-dependent fluctuations in *p*H reported by the dye are complex, and we carried out analytic analysis to better understand it. The noise in photon counts emitted by the dye arises from fluctuations in the protonated number of dye molecules, which follows from binomial statistics as well as photon shot noise, which we assumed to be Poisson distributed in our DS model. As derived in the supporting information in S1 File, applying the transformation in Eq 18, we arrive at the expression for the standard deviation in *p*H^*hν*^ expected from the model:

$$\begin{array}{c} \sigma \left(p{\text{H}}^{h\nu }\right)=\underset{\left|\frac{\partial p\text{H}}{\partial f}\right|}{\underset{︸}{\frac{1}{\text{ln}\left(10\right)f\left(1-f\right)}}}\underset{\sigma \left(f\right)}{\underset{︸}{\sqrt{\frac{f\left(1-f\right)+f/\lambda }{N_{dye}}}}}, \end{array}$$ (19)

where $f=\left[B_{2}\right]/\left[B_{T}\right]=1/\left(1+10^{pH-pK_{a}^{2}}\right)$ is the fraction of protonated dye molecules, λ is the mean photon production count per protonated dye molecule per collection time, and *N*_*dye*_ is the total number of buffer molecules. While this mathematical expression fits our simulated data very well, it also makes it clear that there is no relationship between the fluctuations in the free proton count and the experimental fluctuations in the measured *p*H—the former obeys Poisson statistics and the later is dominated by binomial statistics of the bound dye (Fig B in S1 File). Additionally, the covariance between the number of free protons and the number of bound dye molecules is very weak.

Next, we calculated the noise in the STARs to attempt to validate Eq 19. To do this, we carried out a denoising procedure to extract the fluctuations around the pre-valinomycin bath *p*H, and around steady-state acidified regions from over 100 single vesicle, single transporter traces. First, we identified by visual inspection a set of vesicle traces which remain acidified for an extended period. Next, we used tvdip.m [25], a total variation denoising [26] routine, to aid in selecting a steady-state time window from the most acidified region of each trace. We rejected traces, or narrowed time windows, which contain *p*H spikes consistent with transient pump off states. Finally we selected the subset of traces which acidify to 5.71 < *p*H < 5.81, as this 0.1 *p*H window contained more traces (15) than any other 0.1 *p*H window. We then calculated the Fano factor [27] (the variance divided by the mean) of the raw photon count for these 15 traces both in the steady-state acidified region, and also at *p*H 6.5 using the first 84 data points from before valinomycin addition.

For a Poisson processes, the Fano factor is 1 as the variance equals the mean; however, the fluctuations in raw photon count are super-Poissonian with mean Fano factor values 15—35 (Fig C in S1 File). Additionally, this noise is nearly constant between the two *p*H values. We calculated an expected Fano factor of 20 (see S1 File), in good agreement with the experimental distributions and confirming our prediction that the experimental noise is dominated by the dye and not related to the true *p*H.

### Time-dependent *p*H fluctuations in an acidic compartment

For cellular compartments, there are additional considerations that we have not yet included that could cause deviations from the desired *p*H setpoint, namely the total protein copy number and the realization that pumps—like channels—cycle between on and off states [12]. If during organelle biogenesis a compartment acquires too many or too few proton pumps (or some other critical protein), the pump-to-leak ratio would be skewed pushing the lumen more or less acidic. Likewise, if pumps turn on and off stochastically, like AHA2, then the compartment may experience large fluctuations in time. For eukaryotic cells, the V-ATPase is responsible for acidification [28], and while it is not known if these pumps normally cycle on and off, it is known that they respond to cytoplasmic queues such as low glucose [29], and it is reasonable to assume that they do stochastically inactivate.

**Fig 6:**
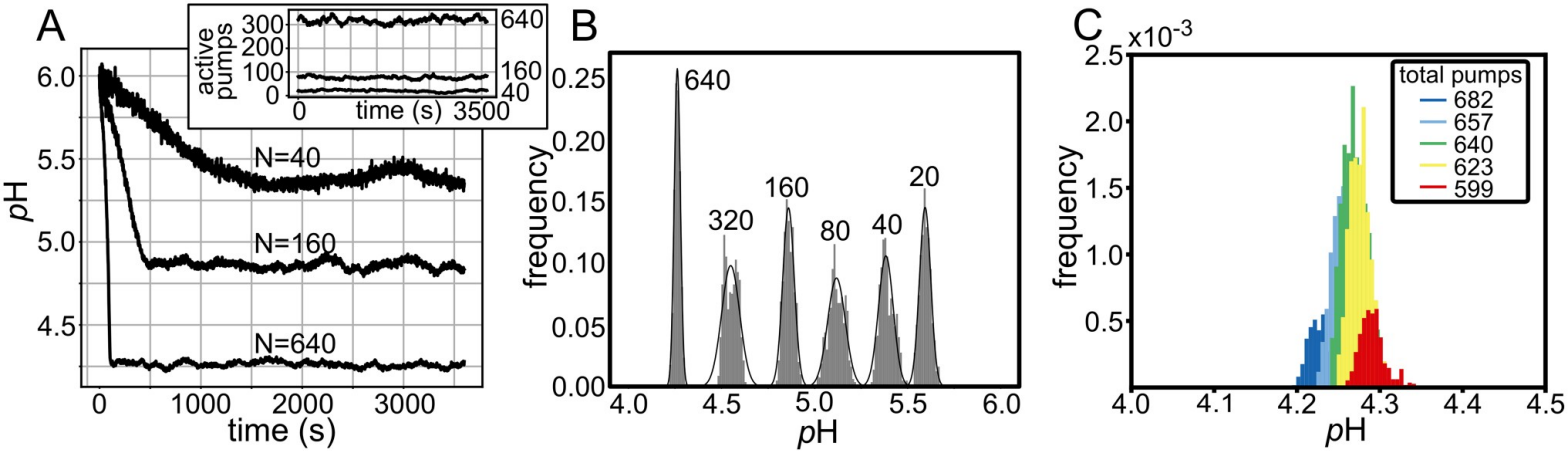
Vesicle *p*H fluctuations are constant over a range of *p*H values. **(A)** DS simulation of a lysosome sized vesicle (*r* = 340 nm), with constant passive membrane permeability *P*_*H*_ = 4.6 × 10^−5^ cm/s, and different numbers of proton pumps *N* = 40, 160, and 640. The pumps stochastically cycle between active and inactive pumping states with a mean dwell time of 273 s in each state and a constant pumping rate of *I*_*P*_ = 100 H^+^/s when active. The inset shows the total active pump population in time. In each simulation, the extracellular *p*H and the starting initial *p*H are 6.0. **(B)** Distribution of *p*H values at steady state for the three simulations in panel A plus three more simulations for *N* = 20, 80, and 320. A normal curve with mean and standard deviation equal to that of each distribution is plotted overtop of each histogram. The *p*H was computed from the instantaneous proton count generated by the DS model in all panels. **(C)** Distribution of steady state *p*H values for a populations of vesicles, as in panel A, with pump numbers pulled from a Poission distribution with mean pump number *N* = 640. The five colors, right to left, correspond to the *p*H values produced by vesicles with the 5^*th*^ (red), 25^*th*^ (yellow), 50^*th*^ (green), 75^*th*^ (cyan), and 95^*th*^ (blue) percentile of the distribution, scaled by their respective probability.

Here, we simulated a 340 nm radius vesicle, which is the typical size of a lysosome [17]. Starting at a *p*H 6, we carried out 6 DS model simulations each with a different number of total V-ATPases ranging from 20 to 640 total pumps (Fig 6). All pumps have the same proton pump rate of 100 H^+^/s [30], but they can each independently activate and inactivate stochastically. For AHA2, the mean dwell time in the on state is 273 seconds, and the off time is comparable under high ATP concentrations [12]. Based on this information, we modeled V-ATPases cycling between active and inactive states by pulling from an exponential distribution with a mean lifetime of 273 seconds for each pump. We were thus able to simulate a population of proton pumps randomly turning on and off, and explore how the steady state *p*H was influenced.

As expected, vesicles with fewer active pumps did not acidify as much as vesicles with far more active pumps (Fig 6A). Initially we thought that as the total pump number (*N*) increased the vesicle would exhibit increasingly smaller noise in the steady state *p*H because the fluctuations in the active pump number scale like $\sqrt{N}$, while the mean number of active pumps scales like *N*. To quantify these fluctuations, we binned the steady state *p*H values produced by the DS model in panel B. While it is true that the vesicle with 640 pumps exhibits the smallest *p*H fluctuations, the width is relatively uniform across the entire range of *p*H setpoints with a total spread of about 0.5 units at neutral values and 0.25 units at acidic values (Fig 6B), which is consistent, but larger, than the more idealized results from Fig 5F. Thus, the other sources of noise in the system, particularly fluctuations in the number of active pumps, contribute to a greater spread in *p*H. Lastly, we wanted to explore one mechanism of *p*H regulation in which there is no active sorting of proton pumps during lysosomal maturation, and N is simply equal to the compartment surface area times the pump density. In this case, the expected number of pumps will be Poisson distributed with a mean N. In order to estimate how an entire ensemble of vesicles would behave, we carried out 5 simulations of a 340 nm radius vesicle with 599, 623, 640, 657, and 682 proton pumps corresponding to 5^*th*^, 25^*th*^, 50^*th*^, 75^*th*^, and 95^*th*^ percentile of a Poisson distribution with mean number of N = 640 pumps, respectively. We then plotted the frequency of observed *p*H values, as in panel B, from each simulation scaled by the probability of observing that number (Fig 6C). As expected, the spread in values is larger than the spread observed from N = 640 alone, but the width is still very small on the order of 0.1 *p*H units.

## Discussion

The process of vesicular acidification is one that is biologically essential, but yet still not completely understood. Mathematical models have been useful in interpreting experiments to help understand how different organelles achieve different *p*H values. The success of past ODE models in particular, in addition to the limitations of these models, motivated us to develop the discrete, stochastic (DS) model of vesicular acidification presented here. The DS model enforces the discrete nature of particles within cellular compartments and describes random fluctuations of *p*H in cellular compartments—two features that are absent in ODE models. We utilized the DS model to estimate the AHA2 proton pump rate and liposome H^+^ membrane permeability from experimental *p*H recordings of isolated vesicles (STARs) from the Stamou Lab. One of the most surprising and important results of our present study is that DS and ODE models provide quantitatively similar parameter estimates (Table 2), suggesting that ODE models are sufficient for describing intracellular ion regulation as long as only mean values are needed and not information regarding the fluctuations.

Measurements of protein and RNA variability have been used to identify unregulated housekeeping genes from Poisson distributed copy numbers [31], to understand how translational efficiency controls noise [32], to reveal how noise can give rise to population variability [33], and to identify how HIV employs a positive feedback loop to control latency [34]. Here, we made an analogy between protein/RNA copy number and proton counts in an attempt to understand the noise in acidic compartments. Unlike many single molecule experiments, the reporter is not the actual tagged molecule of interest, but rather it is related to the number of proton-bound dye molecules. Another difference is that the molecules of interest in gene regulatory network studies are unbounded since the cell can essentially make an unlimited number of molecules, while here the number of reporter molecules is fixed, which means that the expected noise from the reporter is binomial, not Poissonian, and ion fluctuations are not necessarily reflected in fluctuations in the number of bound dye molecules. Therefore, it follows that the fluctuations in *p*H determined from an experimental trace cannot be related back to the true *p*H fluctuations in the vesicle, because the experimental noise is dominated by fluctuations in the bound dye molecules as predicted by Eq 19, which is consistent with experiment (Fig B in S1 File). We also expect that instrumental noise, i.e. from the camera and other sources, will have a small but non-negligible contribution to the fluctuations in *p*H measurements as well, and if this noise is Poissonian with respect to the incident photon count, it will only be about 3-10% of the total noise. One direct consequence of this realization concerning the fluctuations in the dye is that we cannot tell the true range of *p*H values in individual vesicles if these excursions are rather short lived, which may impact our understanding of the range of possible cellular reactions that can take place in a given compartment if its mean *p*H is shifted away from a value needed for catalysis.

Thus, simulation may be one of the best ways to assess the true range of *p*H fluctuations in isolated compartments. Nonetheless, fluorescent reporters do predict the mean *p*H in the compartment, and when averaged over times that are sufficiently long compared to the fastest proton dynamics (e.g. hopping on and off of buffer molecules), experimental changes in mean *p*H are valid and interpretable in terms of our ODE and DS models. In our simple system, the variance in the *p*H decreases with vesicle size and mean *p*H (Fig 5D and 5E), but this trend is less pronounced when multiple proton pumps are present and randomly activated and inactivated. The stochastic switching between on and off states produces single vesicle *p*H distributions that have similar variance across a *p*H range from 4.5-5.6 (Fig 6B). We even considered a population of lysosome-sized organelles of fixed radius whose pump numbers were random, but proportional to the surface area, and the *p*H spread in this ensemble was only about twice the spread observed for a single vesicle (640 pump data in Fig 6B compared to Fig 6C). Thus, based on our initial work here, the relatively large size of the lysosome aids in suppressing noise from stochastic elements in the environment, so that it can achieve an acidic *p*H with a tight distribution.

Now that we have a better understanding of *p*H fluctuations in a simplified system, we intend to build more realistic DS models based on our previous ODE studies of endosomes [15] and lysosomes [17] with a particular interest in exploring how fluctuations in *p*H are influenced by specific channels, transporters, and other molecules localized to each compartment. We will also construct models of synaptic vesicles, which we expect to exhibit large variations in *p*H given their small size. As stated earlier, there is little-to-no experimental evidence for fluctuations or steady state distributions of *p*H in different organelles, and the biological significance of these variations, if they do exist, is scarce. That said, subtle changes in mean *p*H can have a drastic impact on physiology, as exemplified by the recent finding that ClC-7 gain-of-function mutations that results in lysosomal hyperacidifciation by only 0.2 units are associated with delayed myelination, hypopigmentation, and several other adverse effects [35]. Based on examples like this, we expect that compartment-to-compartment variability coupled with ionic fluctuations in time will emerge as important determinants in the homeostatic regulation of key cellular processes, and it will only be a matter of time for the biochemical tools and experiments to mature to the point where these phenomena can be measured and brought to light.

## Supporting information

S1 File Supporting information. Document containing a figure with simulated voltage data corresponding to Fig 3, a mathematical treatment of the fluctuations with two figures, and ODE-based sensitivity analysis with a table.
(PDF)

S2 File Experimental *p*H in Fig 2. Experimental *p*H time series data (in comma separated values format) in Fig 2.
(CSV)

S3 File Experimental *p*H in Fig 3. Experimental *p*H time series data (in comma separated values format) where column 0 is time in seconds and columns 1 through 4 are the *p*H traces 1 through 4 in Fig 3.
(CSV)

S1 Model COPASI model. The discrete, stochastic model created with COPASI. The file format is XML, and it can be opened and run with COPASI [20]. Parameters such as liposome radius, AHA2 pumping rate, pump on and off time, and membrane H^+^ permeability must be set before running the model (Model > Biochemical > Global Quantities). The model is run by selecting the Time Course feature under Tasks.
(CPS)

S2 Model MATLAB ODE model. ODE model of vesicle adicification due to single molecule proton pumping. Description of the inputs can be found in the code. As an example, to reproduce ODE trace #2 from Fig 3, set PH = 3.9; IP = 488; PAHA2 = 65; ph0 = 6.5; radius = 145; pka2 = 6.15; on = 180; off = 506; tfinal = 750; in Matlab (from Table 2), and then run: [t, ph] = S2_Model([PH, IP, PAHA2], on, off, tfinal, ph0, radius, pka2); plot(t, ph).
(M) Click here for additional data file.
