# Supplementary material for: Protons in small spaces: Discrete simulations of vesicle acidification
Source: PLoS Comput Biol. 2019 Dec 23;15(12):e1007539. doi: 10.1371/journal.pcbi.1007539 (PMC6946529; doi:10.1371/journal.pcbi.1007539)
Supplement: S1 File — Document containing a figure with simulated voltage data corresponding to Fig 3, a mathematical treatment of the fluctuations with two figures, and ODE-based sensitivity analysis with a table. (PDF) [file pcbi.1007539.s001.pdf]

Supporting Information

Protons in small spaces: discrete simulations of vesicle acidification

Apeksha Singh[1,2], Frank V. Marcoline[2*], Salome Veshaguri[3,4,5,6], Aimee W. Kao[7], Marcel Bruchez[8,9,10], Joseph A. Mindell[11], Dimitrios Stamou[3,4,5,6], Michael Grabe[2*]

1 College of Letters and Science, University of California, Berkeley, Berkeley, CA 94720, USA
2 Cardiovascular Research Institute, Department of Pharmaceutical Chemistry, University of California San Francisco, San Francisco, California 94158, USA
3 Bionanotecnology and Nanomedicine Laboratory, University of Copenhagen, Copenhagen, Denmark
4 Department of Chemistry, University of Copenhagen, Copenhagen, Denmark
5 Nano-Science Center, University of Copenhagen, Copenhagen, Denmark
6 Lundbeck Foundation Center Biomembranes in Nanomedicine, University of Copenhagen, Copenhagen, Denmark
7 Memory and Aging Center, Department of Neurology, University of California San Francisco, San Francisco, CA 94158, USA
8 Department of Chemistry, Carnegie Mellon University, Pittsburgh, PA 15213, USA
9 Department of Biological Sciences, Carnegie Mellon University, Pittsburgh, PA 15213, USA
10 Molecular Biosensor and Imaging Center, Carnegie Mellon University, Pittsburgh, PA 15213, USA
11 Membrane Transport Biophysics Unit, National Institute of Neurological Disorders and Stroke, National Institutes of Health, Bethesda, MD 20892, USA

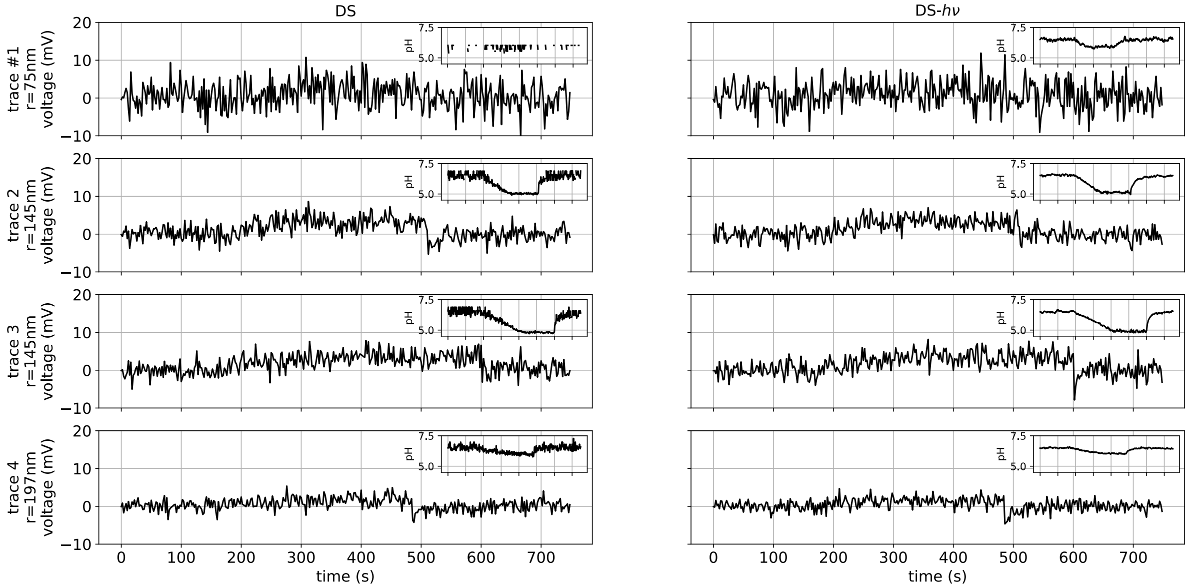

Figure A: **Liposome membrane potential corresponding to DS simulations in Figure 3.** The DS model pH simulation results from Figure 3 are replotted (inset) with the membrane potential (voltage) from each simulation. The voltage is calculated according to Eq. 6 in the main text. The large potassium permeability and high concentrations effectively suppresses the membrane potential during acidification. The fluctuations in membrane potential are increased for smaller sized vesicles, much like pH.

1 Noise in pH measurement from a fluorescent dye

1.1 Shot noise in photon emission rates

Assuming that fluorescence is a stochastic process, the probability $R_{k,d,\lambda}$ of emission of k photons in a unit time interval from d fluorescent molecules, each with the same mean emission rate λ per unit time, is expected to be Poissonian:

$$R_{k,d,\lambda} = \frac{d^k \lambda^k e^{-d\lambda}}{k!}. \tag{1}$$

The expected mean photon count μ is $\mu = \langle k \rangle = d\lambda$, the second moment is $\langle k^2 \rangle = d^2\lambda^2 + d\lambda$, and the variance equals the mean: $\sigma^2 = \langle k^2 \rangle - \langle k \rangle^2 = d\lambda = \mu$.

It has been observed in fluorescent systems that the time between successive photon emissions tends away from zero, a phenomenon known as anti-bunching. Following Mandel [1], we write the variance as $\sigma^2(t) = \mu(t)\,[1 + Q(t)]$, where a $Q(t)$ of zero indicates Poissonian behavior, and $-1 \leq Q(t) < 0$ indicates a sub-Poissonian process. See for example fluorescence in the three-level system of a single Ba^+ ion [2], which exhibits sub-Poissonian statistics. Nonetheless, for the simple treatment below, we assume that photon emission from an ensemble of fluorophores is Poissonian.

1.2 Protonated dye molecule distribution

Consider a dye molecule that only fluoresces when protonated. For a single molecule with a single titratable site, the probability of being protonated is:

$$f = \frac{1}{1 + 10^{p\mathrm{H} - p\mathrm{K}_a}}. \tag{2}$$

For D molecules the probability that d are protonated is given by the binomial distribution:

$$B_{d,D,f} = \binom{D}{d} f^d (1-f)^{D-d}. \tag{3}$$

The relevant moments of the binomial distribution are: $\langle d \rangle = fD$, $\langle d^2 \rangle = f(D - fD + fD^2)$, and $\sigma^2 = \langle d^2 \rangle - \langle d \rangle^2 = f(1-f)D$.

1.3 Noise from a fluorescent dye buffer molecule

For our dye, which only fluoresces when protonated, the expected emission rate for the population is reduced by the mean fraction which is protonated. The probability $P_{k,D,f}$ of emission of k photons per unit time at a particular pH is given by the sum over all dye molecules of the probability of a molecule being protonated multiplied by the photon emission probability for those number of protonated molecules:

$$P_{k,D,f} = \sum_{d=0}^{D} R_{k,d\lambda} B_{d,D,f} \tag{4}$$

The mean expected photon count is:

$$
\begin{aligned}
\mu_\gamma = \langle k \rangle &= \sum_{k=0}^{\infty} k P_{k,D,f} \\
&= \sum_{d=0}^{D} B_{d,D,f} \sum_{k=0}^{\infty} k R_{k,d\lambda} \\
&= \sum_{d=0}^{D} B_{d,D,f} \langle k \rangle_d \\
&= \sum_{d=0}^{D} B_{d,D,f}\, d\lambda \\
&= \langle d \rangle \lambda \\
&= f D \lambda,
\end{aligned} \tag{5}
$$

and the second moment is:

$$
\begin{aligned}
\left\langle k^2 \right\rangle &= \sum_{k=0}^{\infty} k^2 P_{k,D,f} \\
&= \sum_{d=0}^{D} B_{d,D,f} \sum_{k=0}^{\infty} k^2 R_{k,d\lambda} \\
&= \sum_{d=0}^{D} B_{d,D,f} \left\langle k^2 \right\rangle_d \\
&= \sum_{d=0}^{D} B_{d,D,f}\, d\lambda \left(d\lambda + 1 \right) \\
&= \left\langle d^2 \right\rangle \lambda^2 + \langle d \rangle \lambda \\
&= f \left(D - fD + fD^2 \right) \lambda^2 + f D \lambda,
\end{aligned} \tag{6}
$$

The variance in photon count is:

$$
\begin{aligned}
\sigma_\gamma^2 &= \left\langle k^2 \right\rangle - \langle k \rangle^2 \\
&= f \left(D - fD + fD^2 \right) \lambda^2 + f D \lambda - f^2 D^2 \lambda^2 \\
&= f \left(1 - f \right) D \lambda^2 + f D \lambda.
\end{aligned} \tag{7}
$$

From the variance (Eq. 7) and mean (Eq. 5), the Fano factor for the photon distribution is:

$$
F = \sigma_\gamma^2 / \mu_\gamma = (1 - f)\lambda + 1. \tag{8}
$$

1.4 Standard deviation in pH determined by pH sensitive dye

Given a measured mean photon count per unit time $\bar{k}$ at a some known pH, which implies a fraction of protonated dye molecules f, and an estimate of the number of dye molecules

D, you can calculate the mean photon production rate per single protonated dye molecule per unit time λ: $\lambda = \bar{k}/fD$. In practice, if there are other uncorrelated, pH independent noise sources in the system, then $\bar{k} = fD\lambda + c$, where c is the background noise source. Once you determine λ and c from a set of measurement at different pH values, you can determine f, and thus pH, versus k.

In the limit of small c, $f = k/D\lambda$. Then the standard deviation in $f(k)$ is:

$$\sigma_f^2 = \frac{\sigma_\gamma^2}{D^2\lambda^2} = \frac{f(1-f) + f/\lambda}{D}. \tag{9}$$

The standard deviation in pH as a function of the standard deviation in f is then:

$$\sigma_{p\mathrm{H}} = \left|\frac{\partial p\mathrm{H}}{\partial f}\right| \sigma_f \tag{10}$$

Inverting Eq. 2 gives $p\mathrm{H} = p\mathrm{K}_a + \log_{10}(1/f - 1)$, from which we get

$$\frac{\partial p\mathrm{H}}{\partial f} = \frac{-1}{\log(10)\, f(1-f)}. \tag{11}$$

Thus, the standard deviation in pH from the measurement of pH-dependent dye fluorescence is:

$$\sigma_{p\mathrm{H}} = \frac{1}{\log(10)\, f(1-f)} \sqrt{\frac{f(1-f) + f/\lambda}{D}}. \tag{12}$$

In the high photon flux per dye molecule limit ($(1-f) \gg 1/\lambda$),

$$\sigma_{p\mathrm{H}} \to \frac{1}{\log(10)\sqrt{f(1-f)\, D}}, \tag{13}$$

in which case the shot noise contribution to the standard deviation in pH measurement disappears in the region within a couple pH units of the $p\mathrm{K}_a$.

1.5 No contribution from steady-state pH fluctuations in experimental $\sigma_{p\mathrm{H}}$

If protons could only exist as either free or bound to the dye molecule, then the fluctuations in number of protonated dye molecules would be perfectly anticorrelated with fluctuations in the number of free proton. For the single molecule proteoliposome experiments, and the stochastic simulations presented here, the dye buffer is only a small component of the total buffer, and there are many ways to change the free proton count that do not involve protonating a dye molecule. For instance, free protons can enter through the proton pump, leak back out through the membrane, protonate a non-fluorescent buffer, etc.

The top panel of Figure B shows the free proton distribution from a stochastic simulation of a 150 nm vesicle at pH 5. The distribution has a mean of 85 free protons, a variance of 89, and closely resembles a Poisson distribution (Eq. 1) with the same mean.

The bottom panel of Figure B shows the protonated dye distribution for the same simulation, and has a mean of 507 protonated dye molecules out of a total of $D = 604$ for this simulation. The distribution resembles the plotted binomial distribution with the same mean, so that $f = 507/D$ in Eq. 3. The protonated dye distribution is much narrower than the plotted Poisson distribution (Eq. 1) with the same mean.

Fluctuations in the free proton count and the protonated dye count are highly uncorrelated ($\rho = -7 \times 10^{-3}$). Thus, there was no need to include contribution from fluctuations in the free proton count to the standard deviation in experimental pH in Eq. 12.

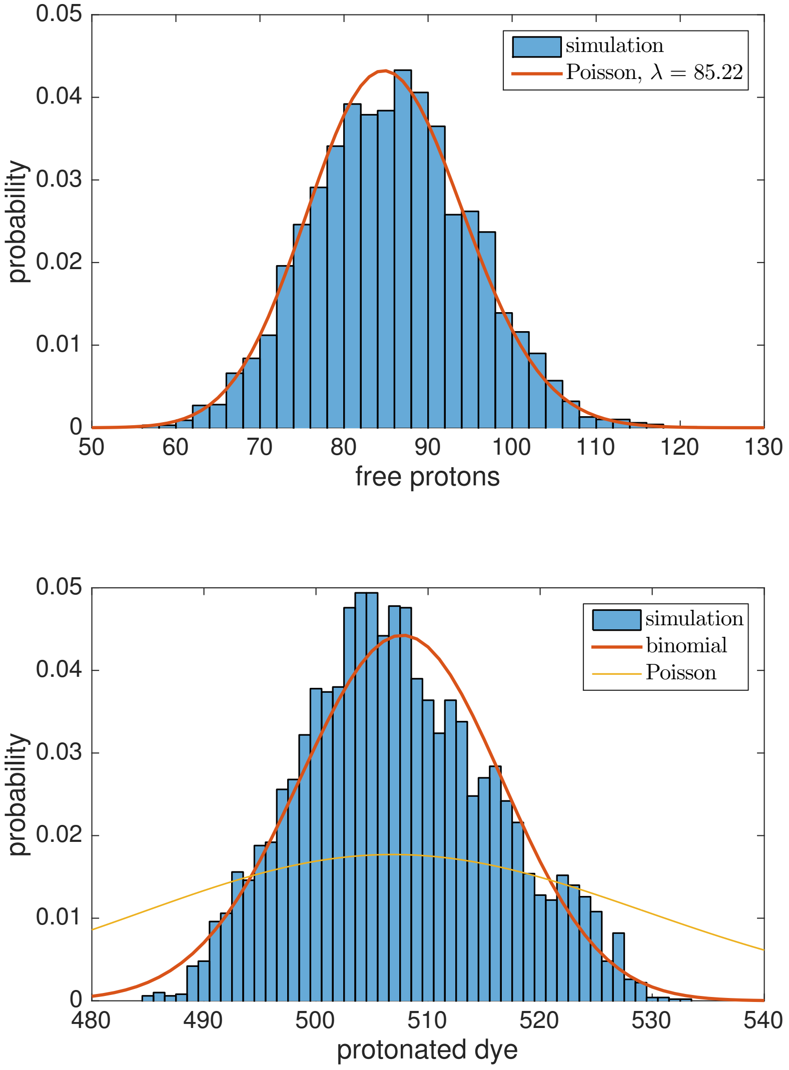

Figure B: **Simulated values of free proton and protonated dye counts. Top panel**: Histogram of free proton count sampled every 0.02 s for 100 s from a steady-state simulation of a 150 nm radius vesicle at a pH of 5, containing a proton pump, a passive proton leak, 85,135 molecules of the primary buffer, and 604 dye molecules. The simulated distribution is consistent a Poisson distribution with a mean of approximately 85. **Bottom panel**: Protonated dye distribution for the simulation in the top panel, shown with the maximum likelihood binomial (Eq. 3) and Poisson distributions (Eq. 1).

1.6 Fano factors of the photon distributions

The Fano factor is defined as the ratio of the variance to the mean in some sample window: $F = \sigma^2/\mu$. As described in the main text, we calculated the experimental F for the photon count from proteoliposomes, which maintained an acidic steady-state pH for an extended time. The theoretical Fano factor of a Poisson distribution is 1, but we find that the Fano factor from these experiments is between 15 and 35, both at the near-neutral bath pH (blue bars top panel) and when acidified (blue bars bottom panel, Fig C). Thus, these experiments are quite noisy, and they do not appear to show strong pH dependence to the noise. Next, we asked whether traces that acidified by the same amount (between pH 5.71 and 5.81) had more tightly clustered Fano factors, and we identified 15 of these traces and plotted their Fano factors computed at both the near-neutral pH (red bars top panel) and acidified values (red bars bottom panel). Again, the distribution was quite wide similar to the entire data set.

Finally, we computed the theoretical Fano factor for these proteoliposomes using $F = (1-f)\lambda+1$ (Eq. 8), with f from Eq. 2 and λ determined from the mean photon count, the dye concentration (Eq. 5) and vesicle volume, using $\lambda = \mu_\gamma/(fD)$ (Eq. 5). This revealed a value of 20, which is in excellent agreement with the values reported in Figure C. Thus, the large Fano factor values are due to the statistics of the dye molecules.

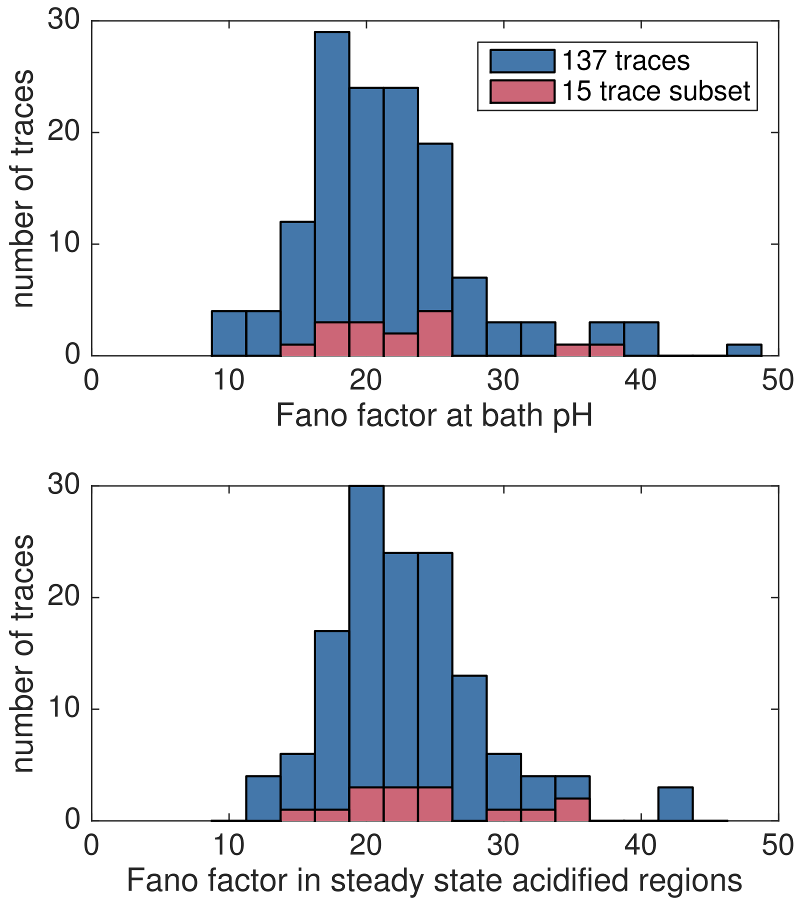

Figure C: **Calculations of experimental noise. Top panel**: Fano factor distribution for photon count from vesicles at bath pH of 6.5 before the addition of valinomycin. **Bottom panel**: Fano factor distribution for photon count for vesicles which maintained an acidified steady-state pH as described in the main text. In both panels, the superposed subset of 15 traces (red) are traces which achieved a steady-state pH between 5.71 and 5.81. Experimental traces taken from Reference [3].

2 Sensitivity analysis of the ODE model

As we have demonstrated, both the discrete, stochastic (DS) and the ordinary differential equation (ODE) models closely reproduce the mean behavior of the single AHA2 proteliposome experiments [3]. Thus, sensitivity analysis of the ODE model is a good proxy for sensitivity analysis of the DS model. Given the clean, well designed experimental conditions, the kinetics of the ODE model are very simple. First, the potassium concentration $[\mathrm{K}^+]$ and permeability P_K are sufficiently large to maintain a near-zero membrane potential (Fig. A). As such, moderate changes ($\pm 10\%$) to $[\mathrm{K}^+]$ and P_K have no significant effect on the vesicle pH as a function of time. Second, the increase in steady-state free proton concentration ($\Delta[\mathrm{H}^+] = [\mathrm{H}^+] - [\mathrm{H}^+]_0$) over the bath concentration $[\mathrm{H}^+]_0$ achieved during proton pumping is a function of the ratio $r = \frac{I_P}{P_H A}$ of the proton pump rate (I_P) to the vesicle proton permeability (P_H) times the vesicle surface area (A). A small percentage change in r results in the same small percentage change in $\Delta[\mathrm{H}^+]$. The rate of acidification during proton pumping is very nearly linearly related to the the concentration of the main buffer species ($[B_1]$), but the extent of acidification is unchanged by changes in $[B_1]$ in the experimental regime in which the main buffer concentration $[B_1]$ is much larger than the dye concentration $[B_2]$. The acidification rate is non-linearly related to $p\mathrm{K}_a^1$, the $p\mathrm{K}_a$ of the main buffer, but again we find that the extent of acidification is nearly independent of $p\mathrm{K}_a^1$. Finally, since $[B_2] \ll [B_1]$, changes in the dye concentration and $p\mathrm{K}_a$ have a negligible effect on the pH versus time.

For this sensitivity analysis, we look at variations in pH using the ODE trace 2 parameters given in Table 2. This trace starts at $p\mathrm{H}_0$ 6.5 and acidifies to a mean pH of 5.10 before the pump deactivates and the vesicle realkalinizes to pH 6.5. The results of varying the parameters discussed above are summarized in Table A.

Table A: **Sensitivity analysis.**

parameter	symbol	default	change	Δ[H$^+$]	ΔpH	τ
pump rate [s^{-1}]	I_p	448	-10%	-10%	+0.04	+9%
			+10%	+10%	-0.04	-8%
H^+ perm. [cm/s]	P_H	3.9×10^{-5}	-10%	+11%	-0.04	+1%
			+10%	-9%	+0.04	-2%
surface area† [nm^2]	A	2.6×10^5	-10%	-11%	-0.04	-13%
			+10%	+9%	+0.04	+13%
K^+ perm. [cm/s]	P_K	10^{-7}	-10%	$\sim$0	$\sim$0	-0.4%
			+10%	$\sim$0	$\sim$0	+0.4%
K^+ conc. [mM]	[K$^+$]	100	-10%	+0.3%	-10^{-3}	-0.4%
			+10%	-0.3%	$+10^{-3}$	+0.4%
buffer conc. [mM]	$[B_1]$	10	-10%	+0.3%	-10^{-3}	-10%
			+10%	-0.3%	$+10^{-3}$	+10%
dye conc. [mM]	$[B_2]$	0.07	-10%	$\sim$0	$\sim$0	$\sim$0
			+10%	$\sim$0	$\sim$0	$\sim$0
buffer pK$_a$	pK$_a^1$	6.1	-0.5	-0.2%	$+10^{-3}$	-13%
			+0.5	+1.2%	-5×10^{-3}	-30%
dye pK$_a$	pK$_a^2$	5.72	-10%	$\sim$0	$\sim$0	$\sim$0
			+10%	$\sim$0	$\sim$0	$\sim$0

The column labeled Δ[H$^+$] is the fractional change in total acidification (initial to final acidified state) due to parameter modification. ΔpH is the change in the amount of acidification due to the variation. The column labeled τ is the fractional change in the $1/e$ decay time from bath pH to fully acidified. The buffer concentration $[B_2]$ is based on the vesicle volume and the mole fraction of lipid which is dye-conjugated, and is thus different from vesicle to vesicle.

† The surface area and volume were both manipulated assuming a spherical geometry.

References

[1] Mandel L. *Sub-Poissonian photon statistics in resonance fluorescence.* Optics Lett. 4(7):205-207, 1979.

[2] Schubert M, Siemers I, Blatt R, Neuhauser W, and Toschek PE. *Photon Antibunching and Non-Poissonian Fluorescence of a Single Three-Level Ion* Phys. Rev. Lett. 68(20), 3016-3019, 1992.

[3] Veshaguri S, Christensen SM, Kemmer GC, Ghale G, Møller MP, Lohr C, Christensen AL, Justesen BH, Jørgensen IL, Schiller J, Hatzakis NS, Grabe M, Pomorski TG, Stamou D. *Direct observation of proton pumping by a eukaryotic P-type ATPase.* Science 351(6280):1469-1473, 2016.
